# The Impact of Curcumin on Growth Performance, Growth-Related Gene Expression, Oxidative Stress, and Immunological Biomarkers in Broiler Chickens at Different Stocking Densities

**DOI:** 10.3390/ani12080958

**Published:** 2022-04-08

**Authors:** Mona H. Hafez, Sara E. El-Kazaz, Badr Alharthi, Heba I. Ghamry, Mohammed A. Alshehri, Samy Sayed, Mustafa Shukry, Yasser S. El-Sayed

**Affiliations:** 1 Physiology Department, Faculty of Veterinary Medicine, Alexandria University, Alexandria 22758, Egypt; 2 Department of Animal Husbandry and Animal Wealth Development, Faculty of Veterinary Medicine, Alexandria University, Alexandria 22758, Egypt; saraelkazaz@alexu.edu.eg; 3 Department of Biology, University College of Al Khurmah, Taif University, P.O. Box 11099, Taif 21944, Saudi Arabia; b.harthi@tu.edu.sa; 4 Department of Home Economics, College of Home Economics, King Khalid University, P.O. Box 960, Abha 61421, Saudi Arabia; hgmry@kku.edu.sa; 5 Biology Department, College of Science, University of Tabuk, Tabuk 71491, Saudi Arabia; ma.alshehri@ut.edu.sa; 6 Department of Science and Technology, University College-Ranyah, Taif University, P.O. Box 11099, Taif 21944, Saudi Arabia; samy_mahmoud@hotmail.com; 7 Physiology Department, Faculty of Veterinary Medicine, Kafrelsheikh University, Kafrelsheikh 33516, Egypt; 8 Department of Forensic Medicine and Toxicology, Faculty of Veterinary Medicine, Damanhour University, Damanhour 22511, Egypt; elsayed-ys@vetmed.dmu.eduu.eg

**Keywords:** broilers, curcumin, physiological efficiency, poultry welfare, stress

## Abstract

**Simple Summary:**

The primary goal of global poultry production is to optimize the amount of chicken produced per square meter of floor area. Consequently, stocking density (SD) and curcumin supplementation on broiler performance were investigated. Our results revealed that supplemental curcumin improved birds’ growth, behaviours, and immunity by lowering oxidative stress, enhancing humoral immune response, and modulating the suppression of growth-related gene expressions in broilers raised in high stocking density circumstances.

**Abstract:**

Curcumin’s antioxidant properties reduce free radicals and may improve broiler growth. Therefore, the influence of stocking density (SD) and administration of curcumin in the diet on broiler performance was explored to clarify the impact of HSD and curcumin on the performance of growth, behavioural patterns, haematological, oxidant/antioxidant parameters, immunity markers, and the growth-related genes expression in broiler chickens. A total of 200 broiler chickens (Cobb 500, 2-weeks old) were allotted into 4 groups; SD (moderate and high) and curcumin (100 and 200 mg/kg diet)-supplemented HSD, respectively. Behavioural observations were performed. After a 28-day experimental period, tissue and blood samples were collected for analysis. Expressions of mRNA for insulin-like growth factor-1 (IGF-1), growth hormone receptor (GHR), myostatin (MSTN), and leptin in liver tissues were examined. HSD birds exhibited lower growth performance measurements, haematological parameters, circulating 3,5,3-triiodothyronine and thyroxine levels, antioxidant activities (GSH-Px, catalase, superoxide dismutase), immunoglobulins (A, G, M), and hepatic GHR and IGF-1 expression values. However, HSD birds even had an increment of serum corticosterone, malondialdehyde, pro-inflammatory cytokine (TNF-a, IL-2, IL-6) levels, hepatic leptin and MSTN expression. Moreover, HSD decreased drinking, feeding, crouching, body care, and increased standing and walking behaviour. The addition of curcumin, particularly at a 200 mg/kg diet, alleviated the effect of HSD through amending growth-related gene expression in the chickens. In conclusion, curcumin can enhance birds’ growth performance, behavioural patterns, and immunity by reducing oxidative stress and up-regulating the growth-related gene expressions of broilers under stressful conditions due to a high stocking density.

## 1. Introduction

The main target of worldwide poultry production is to maximize the bulk of chicken made in each floor space square meter. To gain an appropriate advantage, overloading-related manufacturing damages have been reduced [1]. In the majority of the modern livestock industry, animals’ physiological status is being compromised under several stressful circumstances, such as high rearing density, high ambient temperature, low sanitation, disease challenges, and improper management, thereby threatening health status, productive performance, and the well-being of the animals [2,3]. In addition, stressful circumstances could produce an imbalance between oxygen demand and supply [4]; thus, hypoxia subsequently happens, which enhances the production of free radicals and, as a result, interrupts the normal body functions because of the increased activities of circulating enzymes [5]. Consequently, scientific studies have made numerous efforts to improve the physiological procedures regarding the stress reactions in animals exposed to several stressors.

Stocking density (SD) is considered one of the central alarms in the poultry production industry due to the health consequences and its financial viability. SD is the body’s mass (kg) or the reared quantity of birds in each area (m^2^). High stocking density (HSD) is assumed to decline poultry production due to the high temperature of the environment and decreased flow of air between the bird [6]. An HSD is financially profitable for producers. Recently, HSD has been frequently practised, improving the economic outcomes by gaining other broilers’ meat for each fixed rearing area [2].

The influence of stocking density (20 to 40 kg/m^2^) on broiler production and performance has been studied. However, the majority of these investigations were not always definitive and yielded mixed results. Reduced stocking density has improved broiler performance in some trials [7]. Broiler welfare minimum requirements were announced by the European Commission in 2007, with a maximum stocking density of 30 kg/m^2^ (0.073 m^2^/bird) of broiler chickens across the EU. Furthermore, the National Chicken Council has established a voluntary welfare audit programme for broiler producers. This programme recommends a density range of 31.8 kg/m^2^ for light broilers to 41.6 kg/m^2^ for roosters based on final body weight [8]. Broiler output is heavily influenced by stocking density. Increasing space allowances in the production systems can have a significant negative economic impact on the sector. High-density flocks of birds exhibit physiological adaptations indicative of stress at the transcriptional and telomere levels of the genes they carry [9]. Extension of clutch size experimentally inflicted on the lesser blackback gull [10] has shown that reproductive females deposit more female eggs under dietary stress. This is assumed to be a reproductive adaption. Male gull chicks, more prominent but not heavier at hatching, have fewer reserves and a lower survival rate [11]. In addition, the performance of broiler chickens has been shown to be impacted by the increase in stocking density from 28 to 40 kg BW/m^2^, depending on the system, deep litter or free-range, which could put their welfare at risk [8]. In addition to affecting broiler chickens’ ability to absorb nutrients, the high density also impacts their productivity, the production of stress hormones [4], and their ability to fight off disease [12].

On the other hand, HSD could enhance stress to birds, which is the primary cause of suffering in poultry. This stress induces pathological lesions and deteriorates the broilers’ immune status. On the other hand, broilers need to be delivered ideal environmental circumstances that prompt their genetic perspective.

Li et al. [13] recorded that bodyweight reduces with high stocking density. Nonetheless, HSD has been documented to decline the broiler productivity by decreasing meat quality and growth development and deteriorating the physiological health condition of broilers through the escalating status of oxidative stress [5,14]. There are numerous expensive approaches available to decrease the harmful effects of HSD as outdoor access, but dietary management, through antioxidants supplementation, is a more accessible methodology that is supported by some experimental works [5,14,15].

Numerous experiments on broilers supplemented with phytogenic mixtures of thyme, turmeric, coriander, and others have shown substantial progress in immune indicators, growth performance, and the carcass features in mallard ducks (*Anas platyrhynchos*) [16,17]. Phytogenic mixtures are given individually or in groups as dietary supplements in poultry rations [18]. Among numerous flavours, turmeric (*Curcuma longa* L.), recognized as the golden spice, has exceptional awareness due to its various useful pharmacological properties supporting the well-being and health of poultry and animals [19]. As a member of the Zingiberaceae family, turmeric (*Curcuma longa*) is one of the spices with a member element known as curcumin [20]. Curcumin [1,7-bis (4-hydroxy 3- methoxyphenyl)- 1,6-heptadiene-3,5-dione; diferulylmethane] retains its economic value because of its weird bulb [21]. Curcumin is frequently utilized as a food condiment and a tint due to its defence in contradiction with oxidative stress. It can remove free radicals, guarding tissues and organs, contrary to peroxidation lipids [22]. Furthermore, various experimental and clinical studies have verified that, pharmacologically, curcumin is a harmless substance with antioxidant, anti-inflammatory, and antimicrobial activities [23,24,25,26,27]. It also possesses immune-modulatory and hepatoprotective properties [28].

Nonetheless, the information associated with curcumin usage under HSD circumstances on the physiological responses of broiler birds is scarce. Therefore, the existing work was executed to clarify the impact of HSD and food administration of curcumin on the performance of growth, behavioural patterns, haematological, oxidant/antioxidant parameters, immunity markers, and the growth-related genes expression in broiler chickens.

## 2. Materials and Methods

### 2.1. Experimental Animals

#### 2.1.1. Animal Care

The current experiment was approved and, complying to the rules for maintaining animals and poultry supplied by the National Research Council, was permitted (2021/013/28) by the Resident Commission of Ethics for the Use and Care of Lab Animals at Alexandria University, Egypt. Every phase of the current study plan was performed with insignificant distress or pain for the broilers. The research work of our study was performed at the Department of Animal Husbandry and Animal Wealth Development, Faculty of Veterinary Medicine, Alexandria University. The birds were allocated to ground litter cages with completely controlled temperature and humidity.

#### 2.1.2. Birds and Housing Conditions

The current study was conducted on Cobb 500 chicks (*n* = 200 chicks) from a broilers farm and assigned by weight (to minimize the difference in average body weight) into 4 experimental groups. The ground was cemented and shielded in a fresh straw litter with a depth of 5 cm. The drinking space and food holders were placed so the broilers in every pen acquired approximately identical feeding and drinking area regardless of the stocking density. Broilers were nourished ad libitum consuming a commercial ration. Chicks were fed on starter crumbles, as shown in Table 1 [29], until reaching two weeks of age, and then, for one week, they were provided grower pellets. They were then provided with finisher pellets until the end of the experiment. The temperature of the pens was sustained at 32 °C through the first seven days, and then slowly reduced by 3 °C for each week, until it reached 24 °C by the time the study finished. Controlled artificial light was sustained for 23 h per day during the whole study duration.

MSD (10 birds/m^2^) and HSD (20 birds/m^2^) was accomplished by keeping a different number of broilers for each floor pen [5] with the exact size of the floor (2.0 × 2.4 m²). Before beginning the study, all birds were reared with similar environmental circumstances and were fed similar starter rations for 14 days (Table 1). Space unavailable by permanent substances (i.e., one bell drinker and two feeders) was not involved when the floor space was measured.

At 2 weeks of age, all birds were weighed and divided into 4 groups, including 5 replicates per each group (10 birds/ replicate) for every group of an exact number of birds, as follows: Group 1 (MSD, negative control) (*n* = 50); without curcumin supplementation; Group 2 (HSD, positive control) (*n* = 50); without curcumin supplementation; Group 3 (HSD + curcumin 100 mg kg^−1^ diet) (*n* = 50) [25]; bought from Sigma–Aldrich, St. Louis, MO, USA), curcumin mixed into pellets; Group 4 (HSD + curcumin 200 mg kg^−1^ diet) (*n* = 50) [28].

### 2.2. Growth Performance

Throughout the study progression, broilers were individually weighed weekly (g) to assess the weight gain in body weight, feed conversion ratio (FCR), and to record the intake of food. FCR was measured by dividing the total intake of food (g) by the total gaining of weight (g) of the birds [30] from the beginning of the experiment (14 days old) until the finish of the study (day 42 of age).

### 2.3. Behavioral Observations

The behavioural observation was conducted via continuous visual screening of the flock. The behaviour of all birds was recorded during predetermined time intervals. Experienced behavioural observations were started at two weeks old until the finish of the experiment (6 weeks of age). Observations were undertaken during the daytime (6:00 a.m.–6.00 p.m.) with 12 h of observation divided into 4 h per day through 3 consecutive days. Every day was allocated into two stages, the morning (6:00 a.m.–12:00 p.m.) and the afternoon (12:00 p.m.–6:00 p.m.). The observation was completed for 2 h for every stage of the day, i.e., 2 h during the morning and 2 h during the afternoon with alternation. During day one, the observations were performed from 6:00–8:00 a.m. and 12:00–2:00 p.m.; on day two, it was from 8:00–10:00 a.m. and 2:00–4:00 p.m., and the same for day three [31]. Each observation hour was divided into 5-min intermission scanning of broilers, which was followed with a new 5-min scan of behaviour until the observation time was finished. Behavioural configurations perceived were drinking, feeding, walking, crouching, standing, and behaviour of body care (ruffling, shaking, and preening). The documented behaviours are presented in Table 2. Results were presented as % of broilers doing the categorized behaviour/whole number of broilers scanned [31].

### 2.4. Blood Sampling

On day 42 of age, 6 birds of each replicate were arbitrarily chosen and slaughtered. Two blood samples were taken from the brachial vein, and one was into a heparinized tube for haematological measurements. At the same time, the other sample was collected into plain tubes and centrifuged at 1968× *g* for 15 min. Collected serum samples were retained at −20 °C till examination.

### 2.5. Hematological and Immunological Parameters Measurements

At 1 to 2 h after the collection, the samples of blood were examined for Hb (haemoglobin) level, PCV (packed cell volume), RBCs (red blood cells), and WBCs (white blood cells) count. In addition, a differential leukocytic count was completed. Erythrocyte sedimentation rate (ESR) and RBC settlement rate at uncoagulated blood during an hour inside the Wintrobe tube were then performed [32]. Heterophil/lymphocyte (H/L) ratio was determined by dividing the number of heterophils by lymphocytes. Immunoglobulins in the serum (IgG, IgA, and IgM), pro-inflammatory cytokines (IL-2, IL-6), and tumour necrosis factor-A (TNF-α) were estimated via kits obtained from Elabscience Biotechnology Co., Ltd. (Houston, TX, USA) using a reader for enzyme-linked immunosorbent assay (ELISA).

### 2.6. Biochemical and Hormonal Analysis

Using the specific commercial assay kits (Bio-diagnostic Co., Cairo, Egypt), total serum protein, albumin, total cholesterol, alanine aminotransferase (ALT), and aspartate aminotransferase (AST) were determined using Robert R. GmbH Photometer (5010 VST, Berlin, Germany) [33]. Analysis of thyroid hormones [thyroxine (T4) and triiodothyronine (T3)] and corticosterone concentration according to the photometric recognition principle was accomplished using thyroid hormones and corticosterone ELISA kit (through IBL international GmbH, 22335 Hamburg, Germany).

### 2.7. Serum Malondialdehyde Level and Anti-Oxidative Enzymes Activity

Serum malondialdehyde (MDA) value and superoxide dismutase (SOD) activity were determined using kits supplied from Ransod Diagnostics (London, UK). Meanwhile, glutathione peroxidase (GPx) and catalase (CAT) enzymatic activities were determined via provided test kits (Bio-Diagnostic Co., Cairo, Egypt) following the manufacturer’s instruction.

### 2.8. Gene Expression Analysis

The liver samples (approximately one cm^3^) (*n* = 10) were gathered, then positioned in liquid nitrogen, and kept at −80 °C until investigation. According to the manufacturer’s procedures, total RNA was isolated with TRI reagent (easyRED™, iNtRON Biotechnology, Seongnam-Si, Korea). Its quality was confirmed by gel electrophoresis with 2% agarose. RNA was measured via Nanodrop (UV–VIS Spectrophotometer Q5000, Qua-Well, San Jose, CA, USA). RNA was conversely transcribed to the first-strand cDNA by the SensiFAST™ cDNA synthesis kit (Bioline, London, UK) and kept at −20 °C until the examination. Sequences of gene-specific primers (Table 3) were utilized to strengthen the designated genes. Primer sequences were definite via UCSC In Silico PCR (https://genome.ucsc.edu/cgibin/hgPcr, accessed on 10 March 2021) and NCBI primer blast. Real-time was accomplished by consuming the Stratagene MX300P Realtime PCR machine (Agilent Technologies, Santa Clara, CA, USA), with SensiFast™ SYBR Lo-Rox kit (Bioline, London, UK) according to manufacturer’s recommendations. The intensification technique includes denaturing at 95 °C for 15 min, then by 40 sequences at 95 °C for 15 s and an annealing temperature (specific for every primer, Table 2) for 1 min [15]. Dissociation curves were inspected to approve that only one peak was recognized for each exact melting temperature, therefore presenting that PCR products were propagated specifically. Samples were examined in replicas. Target gene Ct levels were primarily standardized in contradiction of the Ct levels of the house-keeping gene (β actin), then utilized to estimate the gene expression levels (fold change) [34].

### 2.9. Statistical Analysis

Data collected were subjected to ANOVA by applying the General Linear Model Procedure of SAS software (SAS Institute Inc., Cary, NC, USA) [35]. Shapiro-Wilk and Levene tests confirmed normal distribution and homogeneity of variance [36]. Tukey’s multiple comparison test is based on *p* < 0.05. The following formula was exhausted for the examination of variance:

Statistical model

Xij = *μ* + Ti + eij,

where, Xij is the value of ith observation (variables as weight of the body or behaviour or biochemical measurements) of ith treatment, *μ* is the overall mean, Ti is the effect of the treatment (treatment: four different treatments with two stocking density)), and eij is the random error.

## 3. Results

### 3.1. Productive Performance

In Table 4, the HSD group revealed a marked decrease (*p* < 0.0001) in body weight, weight gain, and the intake of food, and an increase in feed conversion ratio (FCR) than the MSD control group. Meanwhile, curcumin with HSD shows a significant increase in body weight, weight gain, and food intake, with a substantial decrease in FCR. However, the higher dose of curcumin (200 mg) displays a significant increase in all productive parameters compared to a lower dose of curcumin (100 mg) as the best bodyweight, weight gain, intake of food, and FCR were observed in the MSD control birds and in the group supplemented with 200 mg of curcumin.

### 3.2. Behavioural Observation

**Figure 1:**
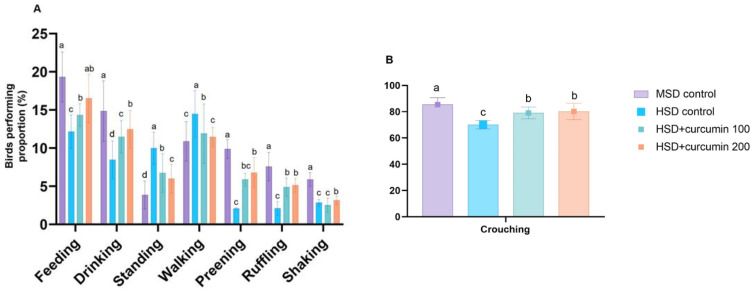
Effect of curcumin on the proportion (%) of birds performing different behavioural patterns (**A**). Feeding, Drinking, Standing, Walking, Preening, Ruffling and Shaking. (**B**). Crouching reared under high stocking density. The data shown are the mean and standard deviation. ^a–d^ Means bearing different superscript letters within the same row are significantly different (*p* < 0.05). MSD = low stocking density, HSD = high stocking density.

In Figure 1, HSD markedly (*p* < 0.0001) reduced the ingestive behaviour of birds. It is considered an allelomimetic behaviour, as two or more birds showed similar behaviour simultaneously with some degree of mutual stimulation and coordination. Therefore, the results revealed a pronounced influence of stocking density on feeding and drinking of engaged broilers. Moreover, crouching and body care behaviour (preening, ruffling, shaking) significantly decreased in birds reared in HSD. In contrast, birds stocked in high density show a significant increase in walking and standing behaviour.

Supplementation of curcumin powder at (200 mg) shows a significant enhancement in the proportion of birds that exhibit standard behaviour patterns than the low dose (100 mg). It was observed that there was a marked enhancement in ingestive behaviour, crouching, and body care behaviour, while there was a substantial decrease in walking and standing behaviour. In comparison, curcumin supplementation shows a considerable enhancement in exhibiting standard patterns of birds’ behaviour.

### 3.3. Haematological Parameters

In Table 5, Hb, PCV, and RBCs count were significantly (*p* < 0.05) decreased in the HSD reared birds relative to their levels in the MSD birds. However, the H/L ratio and ESR were enhanced markedly in the HSD group comparable to the MSD one. However, a marked (*p* < 0.0001) improvement was documented in curcumin (100 or 200 mg/kg diet)-treated HSD chicks Hb, PCV, and RBCs count, and a marked decrease in ESR and H/L ratio relative to HSD-reared broilers. The better values were recorded in a dose-dependent effect. In contrast, the WBCs count revealed a non-significant difference between the groups.

### 3.4. Immunological Parameters

Treatment of the HSD broilers with curcumin (100 or 200 mg/kg diet) led to marked (*p* < 0.05) improvement in IgG and IgA concentrations compared to the HSD group. On the other hand, the HSD broilers showed a significant (*p* < 0.05) reduction in IgA, IgM, and IgG compared to the MSD reared broilers. Moreover, IL-2, IL-6, and TNF-a revealed a marked enhancement (*p* > 0.05) with HSD birds relative to MSD ones. Simultaneously, treatment with curcumin reduced the pro-inflammatory cytokines values to nearly average physiological importance of the MSD group (Table 6).

### 3.5. Biochemical and Hormonal Analysis

The data presented in Table 7 revealed that total serum protein and albumin levels were not varied between the groups. Relative to MSD birds, total cholesterol, ALT, and AST values were markedly enhanced with HSD broilers; however, these parameters were dramatically (*p* < 0.05) decreased with curcumin-treated HSD birds. Relative to 100 mg curcumin kg^−1^ diet HSD treated broilers, the 200 mg curcumin kg^−1^ diet treated birds markedly (*p* < 0.05) enhanced the alternations of these measurements.

As revealed in Table 8, relative to the MSD-raised broilers, the HSD chickens expressed significantly (*p* < 0.05) enhanced corticosterone contents and decreased T3 and T4 levels. Curcumin treatment significantly reduced corticosterone and enhanced T3 and T4 serum levels close to the HSD birds. The 200 mg curcumin/kg diet-treated HSD broilers nearly reached their respective MSD values.

### 3.6. Serum Oxidant/Antioxidant Measurements

In addition, Table 8 showed that curcumin supplementation with the HSD groups markedly (*p* < 0.05) improved SOD, GPx, and CAT activities in birds relative to the HSD broilers. However, MDA was decreased significantly (*p* < 0.05) with curcumin-treated groups close to the HSD broilers. Also, better values were recorded with the 200 mg curcumin/kg diet-treated HSD birds. However, the HSD birds revealed a marked (*p* < 0.05) reduction in SOD, GPx, and CAT activities and a marked increase in MDA relative to MSD-reared birds.

### 3.7. Gene Expression of Growth Regulatory Factors

**Figure 2:**
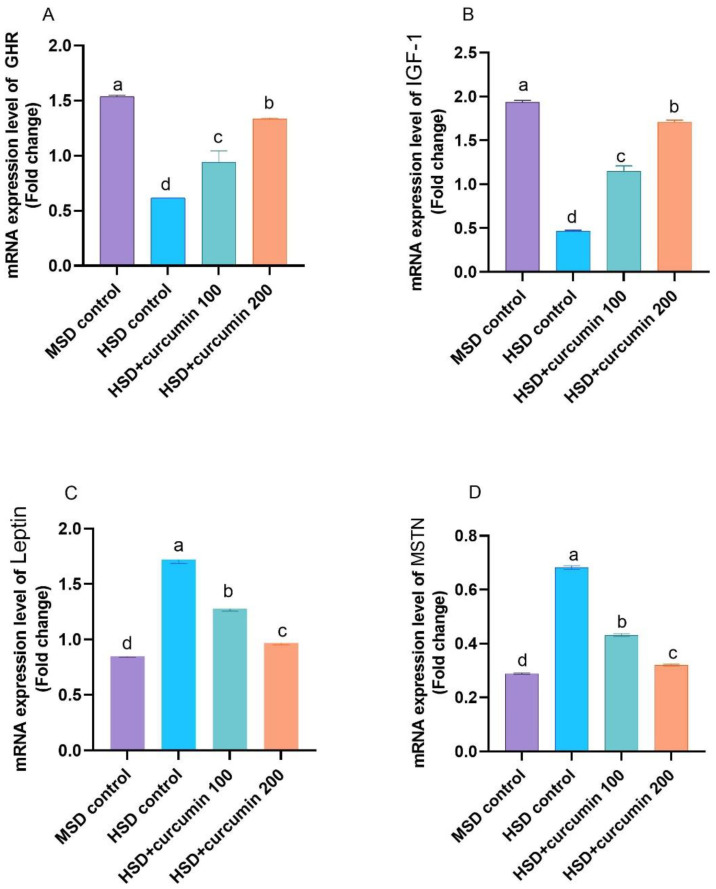
The relative mRNA expression of growth-related regulatory factors of broilers-reared under high stocking density. MSD = low stocking density, HSD = high stocking density, GHR = growth hormone receptor, IGF-1 = insulin-like growth factor 1, MSTN = myostatin. ^a–d^ Columns Means bearing different superscript letters are significantly different (*p* < 0.05).

Growth regulatory genes, IGF-1, and GHR mRNA expressions were downregulated with the HSD broilers (*p* < 0.05) relative to the MSD birds (Figure 2). In contrast, curcumin treatment (100 or 200 mg kg^−1^ diet) with the HSD up-regulated the mRNA expression of GHR and IGF-1 genes (*p* < 0.001); in addition, gene expression of GHR and IGF-1 up-regulation revealed a dose-dependent impact. HSD significantly increased the expression of MSTN and leptin genes relative to the MSD control group. However, curcumin HSD treated groups markedly decreased leptin and MSTN (*p* < 0.001) close to HSD birds; on the contrary, the better values of growth regulatory genes were obtained by 200 mg curcumin kg^−1^ diet treated HSD group.

## 4. Discussion

The existing experiment studied the haematological, immunological, behavioural, and growth performance combined with HSD for broilers given a control ration and two curcumin supplemented diets: 100 and 200 mg/kg. The growth performance with HSD broilers was markedly reduced, and FCR was enhanced relative to MSD birds. In contrast, the curcumin-treated HSD birds were more similar to the MSD group parameters. Similarly, HSD significantly decreased growth performance and compromised intestinal morphology, and metabolized energy results were recorded [5]. Yadav et al. [39] revealed no variation in the growth performance throughout the starter stage of age (0–21 d) with curcumin supplementation at given concentrations.

Nevertheless, broilers served curcumin-supplemented diets for 42 days had a marked enhancement in the body’s weight and food conversion efficiency through a finisher phase (22–42 d). The increased gastric digestion liquor, intestinal villi length, caecal width, and bile production may have improved the digestion of fats. All these benefits are recorded by feeding curcumin for a longer time, enhancing the absorption of nutrients in the advanced stage [22]. Besides, the upregulation of pancreatic lipase and increased trypsin, amylase, and chymotrypsin improved intestinal maltase and sucrose activities [21], reflecting mainly in a higher feed intake and digestion FCR in the curcumin-supplemented birds. Moreover, the negative impacts of these findings with the HSD group may be attributed to the higher levels of serum corticosterone revealed in the current study, which indicate an enhanced stressful condition in the broilers. Those findings were comparable to previous data, in which they reported that the density of stockings had increased reduced growth, FCR, and carcass characteristics; induced oxidative stress and immunodeficiency [13]. For example, increasing corticosterone (catabolic hormone) levels markedly decreases the intake of food and body weight gain in chickens [30]. Meanwhile, curcumin supplementation significantly reduces the corticosterone hormone level, which may enhance the productive performance parameters in birds. Stress can impair immunity and egg production in layers by increasing the level of the stress hormone corticosterone [40].

Behavioural observation revealed that HSD leads to a reduced space allowance, reduced drinking and feeding space, enhanced fighting between the broilers, and significantly reduced ingestive behaviour (feeding and drinking) and crouching behaviour. Moreover, increasing stocking density adversely affects body care behaviour (preening, ruffling, shaking) as birds are under stress when exhibiting body care behaviour [30]. In contrast, increasing stocking density led to a significant increase in standing and walking behaviour. These findings show that as stocking density enhanced, the broilers stand more due to the absence of floor area for lying or crouching. Therefore, HSD limits the floor area accessible for broilers to exhibit normal behaviour, putting the birds under stress conditions. Generally, all features of welfare were harmfully impacted at HSD. These findings agree with [31,41,42,43]; they reported that higher stocking densities resulted in a rise in mortality during the rearing phase, an increase in the leg and contact dermatitis and carcass bruising, as well as a decrease in resting, locomotion, and ground pecking patterns. Stocking density harmed several aspects of welfare. Moreover, HSD induces tissue lipid peroxidation in birds, which increases MDA, reduces serum SOD, GPx, and CAT activity [9,11], and increases the ratio of H/L, the level of corticosterone hormone, and stress indicators in the blood [14]. All these physiological changes are reflected in the birds, as they cannot exhibit their standard behavioural patterns, and it also adversely affects the welfare of the birds.

In comparison, curcumin supplementation of the HSD group shows a significant increase in the welfare of birds as it caused a marked increase in ingestive behaviour and body care behaviour. In contrast, it caused a reduction in walking and standing behaviour, as the curcumin has a significant impact on the inhibition of oxidative stress in exposed chickens via increasing antioxidants enzyme activity and reducing the H/L ratio [44] and corticosterone hormonal level. Therefore, HSD increases stress and disruption to the HPA axis, which induces oxidative stress [45].

The variations in the haematological measurements were recorded in research studies on poultry to evaluate the physiological condition of the birds under stressful conditions [23]. Our results approve with earlier results of Goo et al. [46], who recorded a reduction in Hb, PCV, and RBCs count in the blood of chickens under the effect of HSD; which might be due to the deterioration of the overall health condition accompanied by the reduced feed intake and growth rate. In addition, with environmental or physiological stress (due to HSD), the temperature might rise, which may negatively impact birds’ oxidative condition and the process of erythropoiesis [5]. In the current study, the HSD did not alter the count of WBCs, which agrees with the previous findings [47]. Curcumin treatment improved all the haematological parameters, with concurrent improvement in the antioxidant defence system of broiler under HSD. As a result, the erythropoiesis process is usually resumed [21].

In the current study, a significant impact of SD on other inflammatory markers such as H/L ratio and ESR was recorded. Comparable findings were reported in the ratio of H/L and ESR amongst various SD [14], which might indicate a stressful condition inside the broilers and might be the outcome of a mixture of environmental factors and genotypes. The stressors have rapidly decreased or may be due to the anti-inflammatory impact of curcumin [33]. Conversely, the ratio of H/L and ESR was reduced under curcumin treatment.

The immune system is highly impactful to the animal’s health status. The immune system primarily comprises cell-mediated immunity and humoral immunity. The current study’s marked reduction in serum globulin titers (IgG, IgA, and IgM), suggesting that chickens raised at HSD might be immunocompromised. Likewise, Li et al. [13] recorded that HSD inhibited humoral immunity and enhanced the rate of mortality. Furthermore, the IgG, IgA, and IgM level significantly improved with curcumin supplementation with HSD. Those findings propose that SD might disturb the humoral immune response, particularly curcumin, preventing HSD-mediated Ig synthesis deficiency. In the existing work, HSD enhanced the value of pro-inflammatory cytokine IL-6, IL-2, and TNF-a, which stimulated an inflammatory reaction and therefore was not accepted by the birds.

In an earlier experiment, which enhanced the administration of dietary curcumin, a decrease was reported in IL-6 value in broiler’s blood under the stressful condition of high environmental temperature [22]. The pro-inflammatory cytokines increase with any inflammatory response, encouraging acute-phase protein production, i.e., C-reactive protein, which helps restore homeostasis [48]. In addition, variations occurred in quantities of distinct serum proteins, i.e., an enhancement in fibrinogen and globulins with a reduction in albumins, leading to faster ESR and increased H/L ratio. Subsequently, the administration of curcumin to the diet of broilers under HSD reduced IL-2, IL-6, and TNF-a, so the H/L ratio and ESR value decreased additionally, indicating the potential anti-inflammatory properties of curcumin against HSD [23].

It is believed that during normal physiological circumstances, equilibrium between pro-oxidant production and antioxidant defences is essential in living animals. Disproportioning the compensation raises reactive oxygen species (ROS) and persuades lipid peroxidation. An enhanced value of ROS will further increase the oxidation state and harm the component inside the cell [49]. Consequently, the body is prepared to succeed in the oxidation stress by manufacturing antioxidant enzymes to restore the physiological systems. As GPx, SOD, and CAT, these enzymes play a prominent role in antioxidant defence systems [21]. Our findings relate with findings of Li, Zhang et al. [15] and Selvam, et al. [14], who revealed that HSD caused the initiation of the oxidative stress status in birds under the impact of insufficient floor space, reduced air-flow, and the over-crowding of the birds, as was evident by the higher MDA and lower activates of GPx, SOD, and CAT in the bird’s serum. A lipid peroxidation key end product, MDA, is continuously measured as an oxidative stress biomarker [49]. In the present experiment, the MDA level in the HSD bird’s serum was markedly increased compared to that in MSD birds. This outcome might be owed to over-gathering enhancing the competition between the birds and leading to stress, thus causing elevated lipid peroxidation. Likewise, Li et al. [13] revealed that over-crowding increased oxidative damage and higher MDA production. So, it might be proposed that higher oxidative stress might result from reduced antioxidant enzyme activities with an HSD.

Curcumin can inhibit oxidative stress by improving the activities of antioxidants enzymes containing GPx, CAT, and SOD [43], which was also recorded in the current study in a dose-dependent manner. Consequently, a decrease in lipid peroxidation becomes more vital for keeping the regular production of the animal. Parallel to our results, Salah et al. [21] stated that the administration of curcumin in the ration decreased the TBA (thiobarbituric acid) indicator and enhanced the activities of antioxidant enzymes in chickens relative to those fed a standard ration. The presence of phenolic groups in curcumin construction has an essential role in inhibiting oxidative stress. Those groups can eliminate the superoxide ion, hydroxyl free radicals, and nitric oxides [22]. Akbarian et al. [50] recorded the HSD impact on enhanced activities of AST/ALT in broilers. Higher activities of AST/ALT might indicate a progressive injury to the liver cells, followed by enhanced free radicals, leading to liver lipid peroxidation and injury to various organs [5]. On the contrary, dietary supplementation of the HSD group with curcumin decreased activities of ALT and AST in birds, which indicates that the ability of curcumin to scavenge and neutralize free radicals can shield the liver cells in front of any free radical attack [23]. Consequently, reduced activities of liver enzymes in the current experiment might indicate enhanced functions of the liver succeeding curcumin antioxidant impacts, in order to inhibit any damage to the liver cells by free radicals.

A non-significant variation in blood concentrations of total protein and albumin was revealed in our study; on the contrary, there was a marked enhancement in the whole cholesterol level with HSD. The effect of HSD on higher serum cholesterol in the existing experiment agreed with Wang et al. [51]. They recorded that plasma cholesterol levels enhance with higher stress conditions in broilers. We detected that administration of curcumin to diet markedly reduced the total cholesterol in the blood of broilers relative to those fed a regular diet. Other findings on the impact of curcumin on total cholesterol were recorded by others and a reduction in total cholesterol and LDL in Sprague-Dawley rats [52]. HSD negatively impacted blood lipids, which could be consistent with higher corticosterone release in blood circulation. It is a fact that elevated corticosterone levels in the blood stimulate lipolysis and gluconeogenesis [53], clarifying the increased total cholesterol concentrations.

High corticosterone (catabolic hormone) values can impact the growth of bone and markedly decrease the intake of food and body weight gain in poultry [30]. Serum corticosterone concentrations were increased with HSD birds in the present work. Those findings were comparable to Li et al. [13], who revealed that corticosterone values at 42 D were elevated with HSD (15, 20, 25 per m^2^), which might indicate the enhanced stressful condition of broilers. Several types of research have recorded that stressful situations decreased the serum levels of T4 and T3. The thyroid hormones impact nearly every physiological procedure in the organism and are considered essential to improving broilers’ growth [53]. Nevertheless, data on the impact of SD on thyroid hormones in HSD reared birds is limited. On the contrary, curcumin supplementation to the HSD-raised broilers seems to retain the hormonal profile of the birds to nearly their average physiological values, indicating an ameliorative effect of curcumin against the harmful impacts of HSD [16].

Muscular growth is controlled by various elements, including GHR, IGF-1, MSTN, and leptin. IGF-1 contributes to the growth mechanism, and numerous researchers confirmed that IGF-1 controls the growth of skeletal muscle, which is produced under the GH and GHR control [45]. IGF-1 stimulates skeletal muscle hypertrophy and inhibits muscles atrophy [54]. Leptin plays a critical role in regulating the growth of muscles, and it is produced by adipocytes to signal the brain to decrease the intake of food and improve the expenditure of energy. Hence, it harms growth [55]. MSTN is considered a crucial element for the growth and improvement of muscle, and it also performs as an adverse regulator of muscular development. Before birth, MSTN negatively impacts muscular enlargement, and MSTN inhibits muscle hypertrophy in broilers [55]. In existing work, expression values of mRNA of IGF-1 and GHR in the liver samples were markedly reduced compared to the other groups. However, the expression of mRNA values of MSTN and leptin at HSD was enhanced markedly. Those findings propose that HSD could impact muscular differentiation and hypertrophy by controlling the GHR, IGF-I, leptin, and MSTN gene expression. On the contrary, curcumin supplementation in the diet of HSD reared broilers up-regulated the growth-enhancing genes expression (GHR and IGF-1) while it down-regulated the expression of the growth-inhibitory genes (leptin and MSTN), which might indicate a growth-promoting effect of curcumin [55].

## 5. Conclusions

The supplementation of dietary curcumin had a positive impact on the growth performance. The welfare of the HSD stressed birds, through modulation of the growth-related genes GHR and IGF-1 expression, in addition to the mitigative effect on the inflammatory cytokines (TNF-a, IL-2, IL-6) with increased antioxidant activity and the immunity status was accompanied by improvement of the behavioural performance of the HSD stressed birds Therefore, our study exposed the importance of curcumin supplementation in the poultry diet to relieve the stressful conditions and increase the birds’ growth performance and immune status.

## Figures and Tables

**Table 1:** Composition of experimental starter, grower, and finisher diets (g/kg diet) and the calculated chemical analysis of the basal diet.

Ingredients	Diet		
	Starter	Grower	Finisher
Yellow corn	542	558.8	606
Soybean meal (44%)	319	281	253.3
Corn gluten meal (60%)	71	81	48.1
Vegetable oil	29.8	41	54.4
Limestone ^1^	15	15	15
Monocalcium phosphate ^2^	14	14	14
Common salt	3	3	3
Mineral Premix ^3^	1.5	1.5	1.5
Vitamin Premix ^3^	1.5	1.5	1.5
Methionine ^4^	1	1	1
Lysine ^5^	1	1	1
Anti-coccidial ^6^	0.2	0.2	0.2
Antimould ^7^	1	1	1
**Calculated analysis**			
Crude protein (CP, %)	23.1	22.18	19.39
Metabolizable energy (ME, Kcal/kg diet) ^8^	3053	3160.7	3252.6
Calorie/protein ratio ^9^	132.16	142.5	167.7

^1^ Limestone contains 36% calcium. ^2^ Monocalcium phosphate contains 22% phosphorus and 16% calcium. ^3^ Mineral and Vitamin premix was produced by Heropharm and composed (per 3 kg) of vitamin A 12,000,000 IU, vitamin D3 2,500,000 IU, vitamin E 15,000 IU, vitamin K3 1000 mg, vitamin B1 1000 mg, vitamin B2 3000 mg, vitamin B6 1500 mg, vitamin B12 13.3 mg, niacin 30,000mg, biotin 50 mg, folic acid 600 mg, pantothenic acid 10,000 mg, Mn: 60,000 mg, Zn: 50,000 mg, Fe: 30,000 mg, Cu: 4000 mg, I: 300 mg, Se: 100 mg and Co:100 mg. ^4^ DL-Methionine (Produced by Evonic Co. Birmingham, AL, Germany and contain 99% methionine), ^5^ Lysine (lysine hydrochloride contains 98 % Lysine). ^6^ Kill Cox^®^ (produced by Arabian Company for Pharmaceutical Industries, ^7^ Antimould produced by EL-TOBA CO. For Premixes & Feed El-Sadat city Egypt). ^8^ ME calculated according to NRC (1994), ^9^ Calorie/protein ratio = ME Kcal/CP%.

**Table 2:** Ethogram of the recorded behaviours.

Behaviour	Description
Feeding	Pecking at feed-on-feed troughs.
Drinking	Obtaining water from the cup.
Standing idle	The abdomen is not touching the litter, and the bird is motionless with no other behaviour.
Crouching	The bird remains sitting and lying on the litter, looking around or with closed eyes, no other behaviour.
Walking	Take at least two successive steps.
Preening	The bird cleans and aligns its feathers using the beak.
Ruffling	The action of ruffle or shackling all body feathers.
Shaking	The action of body trembling.

**Table 3:** Gene-specific primers sequence used in the experiment.

Gene	Accession No.	Primer	References	Amplicon Size (bp)	Annealing Temp (°C)
Growth hormone receptor (GHR)	NM_001001293	R: AGAAGTCAGTGTTTGTCAGGG F: AACACAGATACCCAACAGCC	[37]	93	60
Insulin-like growth factor-1 (IGF-1)	NM_001004384	R: CTTGTGGATGGCATGATCT F: CACCTAAATCTGCACGCT	[34]	91	60
Leptin	KT970642.1	R: CCAGGACGCCATCCAGGCTCTCTGGC F: TCCGCCAAGCAGAGGGGT	[38]	261	58
Myostatin (MSTN)	NM_001001461	F: TACCCGCTGACAGTGGATTTC R: GCCTCTGGGATTTGCTTGG	[37]	173	58
β-actin	NM_205518	F: ACCTGAGCGCAAGTACTCTGTCT R: CATCGTACTCCTGCTTGCTGAT	[34]	160	60

**Table 4:** Effect of curcumin on productive performance of broilers reared under high stocking density.

	Initial Body Weight (g)	Final Body Weight (g)	Body Weight Gain (g)	Feed Intake (g)	Feed Conversion Ratio (FCR)
MSD control	412.15 ± 15.23	2101.28 ± 165.23 ^a^	1689.13 ± 161.32 ^a^	2785.26 ± 62.52 ^a^	1.65 ± 0.03 ^b^
HSD control	419.96 ± 18.52	1558.23 ± 150.23 ^c^	1118.27 ± 149.25 ^c^	2099.35 ± 38.11 ^d^	1.88 ± 0.05 ^a^
HSD + curcumin 100	429.88 ± 20.14	1723.77 ± 193.42 ^b^	1293.89 ± 185.96 ^b^	2302.67 ± 26.33 ^c^	1.77 ± 0.06 ^ab^
HSD + curcumin 200	428.23 ± 23.18	1989.87 ± 201.78 ^ab^	1561.64 ± 198.23 ^ab^	2623.25 ± 27.27 ^b^	1.68 ± 0.04 ^b^
*P-Tukey*	0.41	<0.0001	<0.0001	<0.0001	<0.0001

The data shown are the mean and standard deviation using ANOVA. ^a–d^ Means bearing different superscript letters within the same row are significantly different (*p* < 0.05). MSD = low stocking density, HSD = high stocking density.

**Table 5:** Effect of curcumin on haematological parameters of broilers reared under high stocking density.

	PCV (%)	Hb (g/dL)	RBCs (10^12^/L)	WBCs (10^9^/L)	ESR (mm/h)	H/L Ratio (%)
MSD control	24.42 ± 0.27 ^a^	12.23 ± 0.04 ^a^	2.49 ± 0.01 ^a^	24.1 ± 0.26	3.15 ± 0.02 ^c^	0.263 ± 0.008 ^c^
HSD control	17.35 ± 0.31 ^d^	8.35 ± 0.08 ^c^	1.92 ± 0.01 ^b^	24.5 ± 0.38	5.52 ± 0.05 ^a^	0.452 ± 0.007 ^a^
HSD + curcumin 100	20.61 ± 0.24 ^c^	10.87 ± 0.06 ^b^	2.11 ± 0.02 ^ab^	23.6 ± 0.32	4.17 ± 0.01 ^b^	0.329 ± 0.006 ^b^
HSD + curcumin 200	22.61 ± 0.22 ^ab^	11.87 ± 0.06 ^a^	2.31 ± 0.03 ^a^	24.2 ± 0.26	3.97 ± 0.01 ^c^	0.289 ± 0.006 ^c^
*P-Tukey*	<0.0001	<0.0001	<0.0001	0.51	<0.0001	<0.0001

The data shown are the mean and standard deviation. ^a–d^ Means bearing different superscript letters within the same row are significantly different (*p* < 0.05). MSD = low stocking density, HSD = high stocking density, PCV = packed cell volume, Hb = haemoglobin, RBCs = red blood cells, WBCs = white blood cells, ESR = erythrocyte sedimentation rate, H/L = heterophil/lymphocyte.

**Table 6:** Effect of curcumin on immune measurements in the serum of broilers reared under high stocking density.

	IgA (g/L)	IgG (g/L)	IgM (g/L)	IL-2 (pg/mL)	IL-6 (pg/mL)	TNF-a (pg/mL)
MSD control	0.062 ± 0.002 ^a^	0.089 ± 0.003 ^a^	0.092 ± 0.005 ^a^	110.14 ± 5.41 ^d^	15.62 ± 0.87 ^d^	69.92 ± 3.54 ^c^
HSD control	0.015 ± 0.003 ^b^	0.034 ± 0.008 ^c^	0.048 ± 0.009 ^d^	166.67 ± 8.11 ^a^	28.58 ± 2.74 ^a^	98.02 ± 7.22 ^a^
HSD + curcumin 100	0.048 ± 0.002 ^ab^	0.055 ± 0.006 ^bc^	0.066 ± 0.007 ^c^	141.67 ± 6.33 ^b^	22.54 ± 1.66 ^b^	77.37 ± 5.81 ^b^
HSD + curcumin 200	0.061 ± 0.003 ^a^	0.065 ± 0.006 ^b^	0.079 ± 0.007 ^b^	122.14 ± 7.27 ^c^	18.54 ± 0.96 ^c^	71.16 ± 4.54 ^c^
*P-Tukey*	<0.0001	<0.0001	<0.0001	<0.0001	<0.0001	<0.0001

The data shown are the mean and standard deviation. ^a–d^ Means bearing different superscript letters within the same row are significantly different (*p* < 0.05). MSD = low stocking density, HSD = high stocking density, Ig = immunoglobulin, IL = interleukin, TNF = tumour necrosis factor.

**Table 7:** Effect of curcumin on biochemical parameters in the serum of broilers reared under high stocking density.

	Total Protein (mg/dL)	Albumin (mg/dL)	Total Cholesterol (mg/dL)	AST (U/L)	ALT (U/L)
MSD control	3.85 ± 0.25	2.205 ± 0.038	120.0 ± 4.8 ^d^	212.3 ± 16.3 ^d^	24.75 ± 5.04 ^b^
HSD control	3.42 ± 0.31	2.045 ± 0.027	159.8 ± 9.04 ^a^	302.8 ± 19.3 ^a^	32.08 ± 4.22 ^a^
HSD + curcumin 100	3.65 ± 0.21	2.155 ± 0.029	139.8 ± 12.14 ^b^	242.3 ± 12.9 ^b^	29.57 ± 3.73 ^ab^
HSD + curcumin 200	3.81 ± 0.18	2.179 ± 0.038	126.8 ± 8.23 ^c^	226.7 ± 14.7 ^c^	25.75 ± 3.04 ^b^
*P-Tukey*	0.27	0.12	<0.0001	<0.0001	<0.0001

The data shown are the mean and standard deviation. ^a–d^ Means bearing different superscript letters within the same row are significantly different (*p* < 0.05). MSD = low stocking density, HSD = high stocking density, AST = aspartate aminotransferase, ALT = alanine aminotransferase.

**Table 8:** Effect of curcumin on hormonal concentrations and oxidant/antioxidant parameters in the serum of broilers reared under high stocking density.

	Hormonal Concentrations			Oxidant/Antioxidant Parameters			
	T3 (ng/mL)	T4 (ng/mL)	Corticosterone (ng/mL)	MDA (µmol/L)	SOD (U/mL)	GPx (U/L)	CAT (U/L)
MSD control	3.57 ± 0.13 ^a^	55.99 ± 3.84 ^a^	2.95 ± 0.081 ^d^	1.45 ± 0.027 ^c^	2.93 ± 0.031 ^a^	109.57 ± 8.64 ^a^	69.57 ± 4.73 ^a^
HSD control	2.03 ± 0.08 ^c^	29.74 ± 1.77 ^d^	5.74 ± 0.027 ^a^	3.02 ± 0.031 ^a^	1.77 ± 0.017 ^c^	62.22 ± 4.18 ^d^	32.08 ± 2.22 ^c^
HSD + curcumin 100	2.76 ± 0.11 ^b^	41.21 ± 2.03 ^c^	4.06 ± 0.065 ^b^	2.14 ± 0.021 ^b^	2.23 ± 0.029 ^b^	83.41 ± 5.04 ^c^	55.57 ± 3.34 ^b^
HSD + curcumin 200	3.17 ± 0.09 ^a^	49.65 ± 2.22 ^ab^	3.26 ± 0.082 ^c^	1.74 ± 0.021 ^c^	2.72 ± 0.031 ^a^	95.49 ± 4.19 ^b^	63.75 ± 3.84 ^ab^
*P-Tukey*	<0.0001	<0.0001	<0.0001	<0.0001	<0.0001	<0.0001	<0.0001

The data shown are the mean and standard deviation. ^a–d^ Means bearing different superscript letters within the same row are significantly different (*p* < 0.05). MSD = low stocking density, HSD = high stocking density, T3 = triiodothyronine, T4 = thyroxine, MDA = malondialdehyde, SOD = superoxide dismutase, GPx = glutathione peroxidase, CAT= catalase.

## Data Availability

Data are available upon request.
